# SLING: a tool to search for linked genes in bacterial datasets

**DOI:** 10.1093/nar/gky738

**Published:** 2018-08-16

**Authors:** Gal Horesh, Alexander Harms, Cinzia Fino, Leopold Parts, Kenn Gerdes, Eva Heinz, Nicholas Robert Thomson

**Affiliations:** 1 Wellcome Sanger Institute, Wellcome Trust Genome Campus, Hinxton, Cambridgeshire, United Kingdom; 2 Centre of Excellence for Bacterial Stress Response and Persistence, Department of Biology, University of Copenhagen, Copenhagen, Denmark; 3 Department of Infectious and Tropical Diseases, London School of Hygiene & Tropical Medicine, London, United Kingdom

## Abstract

Gene arrays and operons that encode functionally linked proteins form the most basic unit of transcriptional regulation in bacteria. Rules that govern the order and orientation of genes in these systems have been defined; however, these were based on a small set of genomes that may not be representative. The growing availability of large genomic datasets presents an opportunity to test these rules, to define the full range and diversity of these systems, and to understand their evolution. Here we present SLING, a tool to **S**earch for **LIN**ked **G**enes by searching for a single functionally essential gene, along with its neighbours in a rule-defined proximity (https://github.com/ghoresh11/sling/wiki). Examining this subset of genes enables us to understand the basic diversity of these genetic systems in large datasets. We demonstrate the utility of SLING on a clinical collection of enteropathogenic *Escherichia coli* for two relevant operons: toxin antitoxin (TA) systems and RND efflux pumps. By examining the diversity of these systems, we gain insight on distinct classes of operons which present variable levels of prevalence and ability to be lost or gained. The importance of this analysis is not limited to TA systems and RND pumps, and can be expanded to understand the diversity of many other relevant gene arrays.

## INTRODUCTION

Operons and functionally linked gene arrays represent the most basic unit of transcriptional organization in prokaryotic genomes (1). Genes involved in the same process or pathway are encoded in a single block, and transcribed under the same regulation (1). Many clinically important gene systems are encoded in operons; all secretion systems (2,3), CRISPR-cas systems (4,5), Resistance Nodulation Division (RND) efflux pumps (6), toxin antitoxin (TA) systems (7,8) and more follow this organization.

The structure of operons and gene arrays with similar function can vary substantially across isolates and species. The order of the genes is often changed, and individual genes may be lost or gained (4,9,10). All of these differences complicate comparisons of these systems between genomes in large datasets. To resolve these issues, sophisticated methods have been developed to annotate specific operons (3,11–14). These tools are restricted to particular operons as they rely on previously defined structures and sequences, or require reprogramming for identification of new genetic structures. Alternatively, tools have been developed to predict all operons in bacterial genomes, and have been used to construct databases (15–18). Many of these tools apply their searches on genome annotation files, leading to systems which remain unobserved as they are not recognised by automatic annotation programmes due to very short coding sequences. With the growing availability of large datasets for the surveillance of important pathogens (19–21), there is a need for a single flexible framework to annotate clinically relevant gene arrays across a range of isolates and examine their diversity.

Here we present SLING, a tool to **S**earch for **LIN**ked **G**enes (https://github.com/ghoresh11/sling/wiki). SLING defines a gene array as a single conserved gene together with its neighbours in a rule-defined proximity and orientation. This definition allows SLING to capture the potential diversity of the gene array across isolates, and allows identifying and studying their variability. For instance, RND efflux operons always contain an RND efflux pump protein, which is often located downstream of the membrane fusion protein (6). In toxin antitoxin (TA) systems, a toxin protein is encoded in close proximity to its cognate antitoxin. Using SLING, we were able to identify and characterise these two operons in an existing example dataset comprised of 70 enteropathogenic *Escherichia coli* (EPEC) genomes taken from (22) and selected *E. coli* reference strain genomes. We gained insights into the distribution of these systems across the isolate phylogeny as well as the variation in their genetic components, identified associations with specific lineages, and obtained a deeper understanding about the pattern of loss or gain of the complete arrays or their components across the phylogeny.

## MATERIALS AND METHODS

### SLING

**Figure 1.**
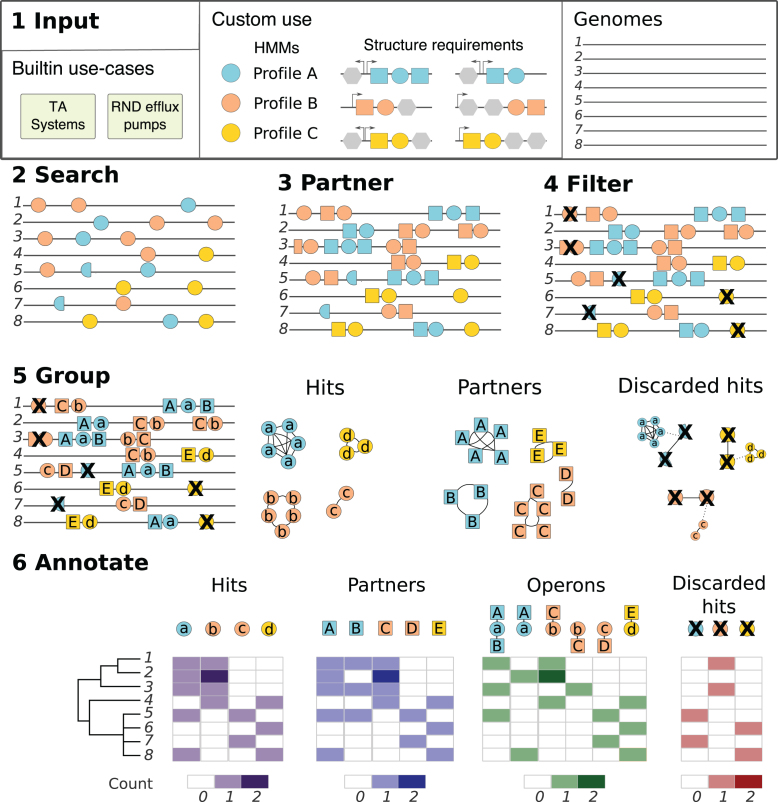
Overview of the SLING pipeline. (1) SLING input. The user may use one of the built-in cases or otherwise provide SLING with a collection of HMM profiles and structural requirements. The structural requirements presented provide a simple example of gene arrays with multiple possible structures (top left). Grey octagons represent variable genes. Circles represent conserved genes each with a matching HMM profile represented by a unique colour which are used in the SLING search. Squares represent the partner genes consistently found in a rule-defined proximity to the conserved gene. (2) HMM profile hits are found in the input genomes. (3) Partner genes are located. (4) Partner genes are filtered based on the given structural requirements. (5) Hits, partners and discarded hits are grouped (alphabetic labelling) using sequence similarity networks. Discarded hits are mapped back to the accepted hits. (6) SLING outputs can be loaded into ITOL for visualisation of results. Phylogenetic tree needs to be provided for visualisation.

SLING is implemented in Python (2.7) and is available to download from https://github.com/ghoresh11/sling. For full details and example use cases, please refer to the package wiki (https://github.com/ghoresh11/sling/wiki). A detailed workflow of the SLING search strategy is given in the Results section (Figure 1).

#### Genome preparation

Complete genomes or assembled contigs in FASTA format were six-frame translated using Biopython v1.68 (23). By default, translation is performed using the standard codon table and the permitted start codons are [ATG, TTG, GTG], i.e. SLING will search for the longest coding sequence (CDS) beginning with ATG, if it is not found it will search for the longest CDS beginning with TTG and finally GTG. Annotation files of the provided genomes in GFF format can also be provided to increase sensitivity of the search (Supplementary Figure S1).

#### Searching

HMMER (v3.1b2) (24) was used to search all CDSs for the profiles of the primary gene provided by the user. The cut off used for a CDS to be considered a ‘hit’ for downstream analysis is a HMMER bit score of the overall sequence/profile comparison of at least 20. The cutoff was chosen based on the scores of toxin HMM profiles in known toxin sequences downloaded from TADB (12,25). For the applications presented in this paper, the HMM profile libraries are built into SLING.

#### Filtering

‘Partner’ genes are searched in proximity to the hits according to structure requirements provided by the user. The structure requirements include the orientation of the partner gene relative to the conserved gene (upstream, downstream or both for a three-component array), the minimum and maximum length of the conserved gene, the minimum and maximum lengths of the partner genes (upstream and downstream if applicable), and the limitations on the location of the partner gene relative to the conserved gene (maximum overlap and distance). If no partner is found under the given requirements, the hit is discarded. For the built-in HMM collections presented in this paper, these requirements are provided by SLING; however, the default values can easily be overridden. Partner genes which have eight or more consecutive unknown nucleotides (Xs or Ns) are removed at this stage and not considered by SLING.

#### Profile-specific length requirements

The user can provide SLING with a file containing the expected length of proteins of each of the profiles in the HMM collection, and a limit on the maximum permitted difference between a hit's length and its expected length. This is useful when scanning for multiple profiles of conserved proteins that have versatile expected lengths.

#### Grouping

Sequence similarity networks (SSN) are constructed for all the hits and the partners identified using protein-protein BLAST+ (v2.7) (26). When using an orientation requirement of ‘either’, SLING will treat upstream and downstream partners the same to form a single SSN. When using ‘both’, SLING will generate an SSN for the upstream partners and the downstream partners separately.

Each node in an SSN is either a hit or partner sequence. An edge is drawn between two hit nodes or two partner nodes only if they meet the minimum requirements of sequence similarity as provided by the user for the BLAST output. The default requirements applied for the results in this paper are an e-value of 0.01 and an identity of 30%. All sequences found in the same connected component in the SSN are considered to be in the same sequence cluster. Each identified gene array is labelled with its relevant hit and partner clusters.

#### Reporting discarded HMM matches

The discarded hit sequences are grouped in an SSN as described above. Each connected component in this network is then mapped back to the clusters in the hits network and the discarded hit clusters are labelled according to their equivalent hit cluster.

### RND efflux pump data preparation

**Figure 2.**
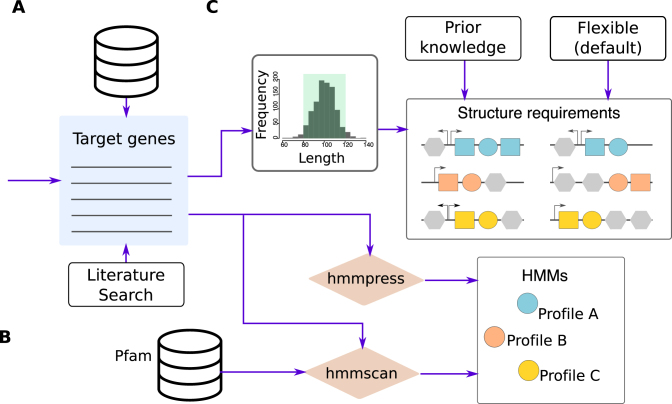
Construction of HMM profiles and structural requirements for SLING input. (**A**) A collection of known target genes is required, taken from existing databases (toxins; TADB, RND pumps; Uniprot), a literature search or other sources. (**B**) HMM profiles can be generated directly from the target sequences using HMMER (24) hmmpress or can be scanned by HMMER hmmscan against existing HMM profile databases, for instance, Pfam (http://eddylab.org/software/hmmer3/3.1b2/Userguide.pdf) (29). (**C**) Structural requirements can be inferred from the target gene sequences, known from prior knowledge or otherwise, flexible using SLING’s default parameters.

3,325 RND efflux pump sequences were downloaded (on 07.11.17) from Uniprot (27) by search of 26 known RND pump genes, taken from 295 different genera (Supplementary Table S1) (Figure 2A). Sequences were clustered using cd-hit (v4.7) to remove redundant sequences which share 90% identity (28). The remaining 1,242 sequences were searched using HMMER (v3.1b2) against the Pfam database (v30.0) to identify known RND pump domains (24,29) (Figure 2B). A total of 29 Pfam profiles were identified in these sequences, of which a single profile, *ACR_tran* (PF00873), was chosen to represent all RND pumps as it was present in over 99% of the sequences.

The length distribution of the above mentioned RND pump proteins were plotted (Supplementary Figure S2A) (Figure 2C). A minimum length of 700 aa long and maximum length of 1500 aa long were chosen for the RND pump protein, covering over 94% of the downloaded sequences. For the partner gene, a minimum length of 100 aa and maximum length of 1000 aa were chosen as flexible requirements for different partner genes and covering the length of over 99% of membrane fusion proteins downloaded (on 07.11.17) from Uniprot (27) (Supplementary Figure S2B). Finally, we allowed a maximum of 500 bp distance between the partner and the RND pump, and at most 20 bp overlap.

### Strains and phylogenetic analysis

The core gene phylogeny of 91 EPEC *E. coli* strains (Supplementary Table S2) was inferred from a core gene alignment generated using Roary (30), and a maximum likelihood tree from the informative SNPs, chosen using SNP-sites (31) (v2.3.2), was constructed using RAxML (v8.2.8) (32) with 100 bootstrap replicates.

## RESULTS

### SLING

SLING is a command line tool which requires a collection of assembled genomes (contigs or complete), Hidden Markov Models (HMMs) representing a conserved gene within the gene array of interest and optional structural requirements as input (Figure 1). HMMs are statistical representations of protein multiple sequence alignments which can be used to search for homologous proteins (33). For the use cases presented in this paper, the HMM profile libraries and structural requirements are built into SLING. Briefly, each HMM profile is used to search the genomes for the presence or absence of the primary gene. If the gene is detected, referred to as a ‘hit’, SLING attempts to identify the partner protein coding sequences proximal to it. The results are filtered to match the provided structural requirements, for example the distance between the partner and hit or their permitted lengths. If the structural requirements are unknown, SLING will search for the closest neighbouring genes with no limitations. Hits, partners and discarded hits are grouped using sequence similarity networks. Finally, SLING reports the number of occurrences of each hit group, partner group, complete array group and discarded hit group found in each genome. These can easily be loaded into statistical analysis tools or into ITOL (34), an online tool for display and management of phylogenetic trees, creating an immediate interface for the user to examine the distribution across large datasets. SLING is available to download from https://github.com/ghoresh11/sling. Full details, including the parameters used in the example applications, are provided in Materials and Methods and in the package wiki (https://github.com/ghoresh11/sling/wiki).

### Applications

#### Toxin antitoxin systems

**Figure 3.**
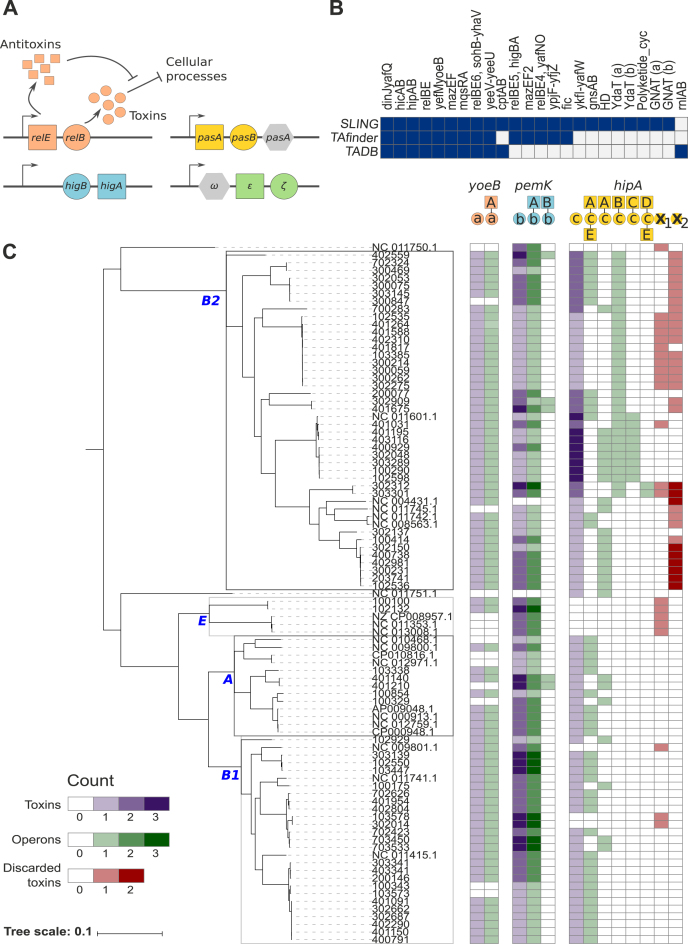
Identification of TA systems using SLING. (**A**) Possible structures of TA operons (7). Each toxin has a unique HMM profile, represented by different colours. (**B**) Identification of TA systems in *E. coli* K-12 using SLING, TADB and TAfinder. Positive prediction by a method represented in a dark blue square. (**C**) Description of three toxins and their cognate antitoxins in the *E. coli* collection.

Toxin Antitoxin (TA) systems were first described to play a role in plasmid maintenance via post-segregational killing of daughter cells that lose the plasmid containing the TA system (35). They have since been found to be ubiquitous in bacterial chromosomes, but their proposed role in other important cellular processes such as persistence, biofilm formation, defence against phage and stress response, is not well understood (8,36). The majority of TA systems are two component operons. One gene encodes a toxin that targets essential cellular processes such as transcription or translation, leading to the inhibition of cell growth or cell death. The second gene is the toxin's cognate antitoxin which inhibits the toxin's activity (7,36) (Figure 3A). Altogether, there are six types of TA systems, defined by the antitoxin and the nature of its interaction with the toxin (36). Here, we focus on type II TA systems in which the cognate antitoxin is a protein that inhibits the toxin through direct interactions. We chose to focus on these systems to show the utility of the program as type II systems are well studied and their structure is generally known; the antitoxin and toxin genes are transcriptionally coupled with well-defined rules describing the gene orientations and distance separating them (7,36). Moreover, an extensive database of type II TA systems, TADB (12,25) was available to us as a resource to benchmark the approach. Following the same set of rules, we have also included type IV systems in which the antitoxin is also a protein which inhibits the toxin's activity via the toxin's target (37). Only a few type IV systems have been described so far, and appear to be rare compared to the abundant type II TA systems (37).

To generate a collection of toxin HMM profiles, used as the primary gene in SLING, type II and type IV toxin sequences were retrieved from the web based resource for toxin-antitoxin loci, TADB (25) and were supplemented by additional toxin sequences based on a literature search (Figure 2A). All the toxin sequences were scanned against the Pfam protein domain database with HMMER to identify known toxin domains, obtaining an initial set of 155 putative domains (24,29) (Figure 2B). Antitoxin domains and domains of non-protein based TA systems were removed as they were not the subject of this investigation. A test dataset of 33 *Klebsiella pneumoniae* genomes and plasmids (38) (Supplementary Table S3) was used to identify and remove Pfam profiles present in a high diversity of non-toxin proteins that could lead to low specificity in identifying toxins (Supplementary Figure S3). The final collection consisted of 54 toxin profiles (see Supplementary Table S4).

The orientation requirement was set based on the knowledge that the partner gene, i.e. the antitoxin, can be either upstream or downstream of the toxin gene (7). The length distributions of the toxin and antitoxin sequences downloaded from TADB were plotted (Supplementary Figure S4) to define the length requirements (Figure 2C). Over 90% of the toxins were between 30 and 200 aa long, and over 90% of antitoxins were between 50 and 150 aa long; therefore, these were used as the relevant cutoffs. Finally, a distance of up to 50 bp and an overlap of at most 20 bp was permitted between the toxin and antitoxin genes. The average length of all the toxin sequences retrieved from TADB containing each of the Pfam domains in our collection was calculated (See Supplementary Table S4). These lengths vary quite significantly for the different toxins. For these profiles, only hits which were up to 100 aa longer or shorter than the average toxin length were accepted for further steps.

A similar process can be applied to construct the HMM profile libraries of other genes and to define the structural parameters. HMM profiles can also be generated directly from a collection of genes using HMMER (Figure 2B) (http://eddylab.org/software/hmmer3/3.1b2/Userguide.pdf) (24). Finally, if the structural requirements are unknown, SLING provides default parameters for a flexible search which will identify the closest partner genes proximate to the primary gene.

##### SLING identifies new and known TA systems in *E. coli* K-12

Using the parameters described above we searched *E. coli* K-12 strain MG1655 (NC_000913.3) for TA systems. SLING identified 23 TA systems in total (Figure 3B, Supplementary Table S5). We compared these results to the TA systems in TADB and those predicted by TAfinder, the search program offered by TADB, using the same parameters used in SLING (12,25). Nine of the 23 systems were identified by all three methods. TADB missed five TA predictions which were identified by the other two methods, whereas TAfinder missed one. A single system, identified by TADB, is missed by both SLING and TAfinder, the *rnlAB* system. The RnlA toxin has a length of 397 aa, beyond the maximum length threshold of 200 aa for a toxin applied in our implementation.

SLING identified eight TA systems which were not predicted by TADB or TAfinder. Of these, four have been predicted in the past to be TA systems; the YkfI-YafW system (37,39), the GnsAB TA system (40), the RatAB system (41) and the YdaST system (42). Four more predictions have not been previously described as TA systems and are candidates for further investigation. One contains an HD domain, two contain a GNAT domain and the last a YdaT toxin domain, consistent with their proposed function.

TADB and TAfinder identified TA systems that were not identified by SLING. Thirteen of the TADB results belonged to the TA system classes that were not investigated in this study (Supplementary Table S5). An additional two toxins were predicted which, using HMMER, did not contain any described toxin profile used by SLING. Finally, TAfinder predicts three TA systems which we attempted to retrieve from the reference genome but were unable to identify complete CDSs at the relevant locus.

##### TA systems present different inheritance patterns and antitoxin repertoire in the EPEC collection

To search for TA systems in a diverse set of related bacteria we applied SLING with standard settings (see Materials and Methods) on a collection of 70 EPEC isolate genomes taken from (22), supplemented by an additional 21 commonly studied *E. coli* reference strains (Supplementary Table S2). The EPEC isolates were collected from children presenting with diarrhea from seven centres in Africa and Asia (22).

SLING identified a total of 94 different TA operons in the complete *E. coli* collection built of 44 toxin (hit) clusters and 80 antitoxin (partner) clusters. The toxins (Supplementary Figure S5), antitoxins (Supplementary Figure S6) and operons (Supplementary Figure S7) are distributed differently along the phylogeny; some are ubiquitous and present in all the isolates, often in more than a single copy, others are specific to one of the *E. coli* phylogroups, and others are rare, present only in a small number of isolates. These results suggest that the diversity of TA systems in *E. coli* is not only driven by the range of toxins, but enhanced by the high diversity of putative antitoxins located in proximity to these toxins.

Below we present examples of three toxins to illustrate the type of analysis and interpretation that can be accomplished using SLING (Figure 3C).

##### YoeB toxin presents low antitoxin repertoire, with low evidence of gene loss/gain

Using the YoeB profile (Figure 3C), *yoeB* was never found as an orphan gene with all genes identified being partnered with the same antitoxin, strengthening the hypothesis that it serves as a toxin in a TA system. This system was also ubiquitous, present across all EPEC phylogroups. In addition, there was no evidence of duplication events, with a single copy of the operon in each isolate. Interestingly there were examples of loss or gain of the whole operon in nine locations within the phylogeny, strengthening the hypothesis that it serves as a toxin in a TA system.

##### PemK toxin presents medium antitoxin repertoire, with high evidence of gene loss/gain

The second toxin (Figure 3C), containing a PemK profile, showed diversity in its antitoxin repertoire: it is found with two different antitoxins: A and B. Most copies of this toxin are found with one of the antitoxins (A; 97%), which is present across all the phylogroups. For this operon, there is a strong indication of gain events followed by fixation and vertical propagation; a subclade with a copy number of *n* is often found within a clade with copy number *n-1*. This phenomenon occurred independently multiple times in the phylogeny. The pervasiveness of this operon can either allude to its importance, or otherwise, suggests it is successful at spreading in the population and persisting. The second operon (B) is rare and found only in five isolates as a single copy. It was most likely acquired in three independent events. Finally, like *yoeB* toxin, this toxin was always recognised as a valid hit by SLING.

##### HipA toxin presents high antitoxin repertoire, with low evidence of gain/loss of the same genes

The third toxin (Figure 3C), containing a HipA profile, presents a much higher diversity in its antitoxin repertoire with five candidate antitoxins. Four of these antitoxins (A–D) are upstream to the toxin, whereas the last antitoxin (E) is found downstream to the toxin and is always present with one of the upstream antitoxins.

Looking at their phylogenetic distribution, although many of the isolates have more than one copy of the *hipA* toxin, it is apparent that within one genome each individual toxin gene is partnered with a different antitoxin. The majority of toxin genes are linked to antitoxin A (62%), which together are present across all phylogroups (Figure 3C). The three other antitoxins (B, C and D) are lineage specific and only present in the B2 phylogroup. Interestingly, all isolates with antitoxins C or D also have antitoxin B.

Although HipA is a well described toxin, we observed many cases in which SLING filtered the predicted toxin gene out due to deviations from the expected operon structure. These genes were marked as discarded by SLING as a result of this. However, analysis of these discarded toxins showed that they formed two separate sequence clusters: *X*_1_ and *X*_2_. All the *X*_1_ toxins coincide with isolates which are missing the A antitoxin. As for *X*_2_, all the discarded toxins are within phylogroup B2, coinciding with isolates which are missing antitoxins B and C.

#### RND efflux pumps

**Figure 4.**
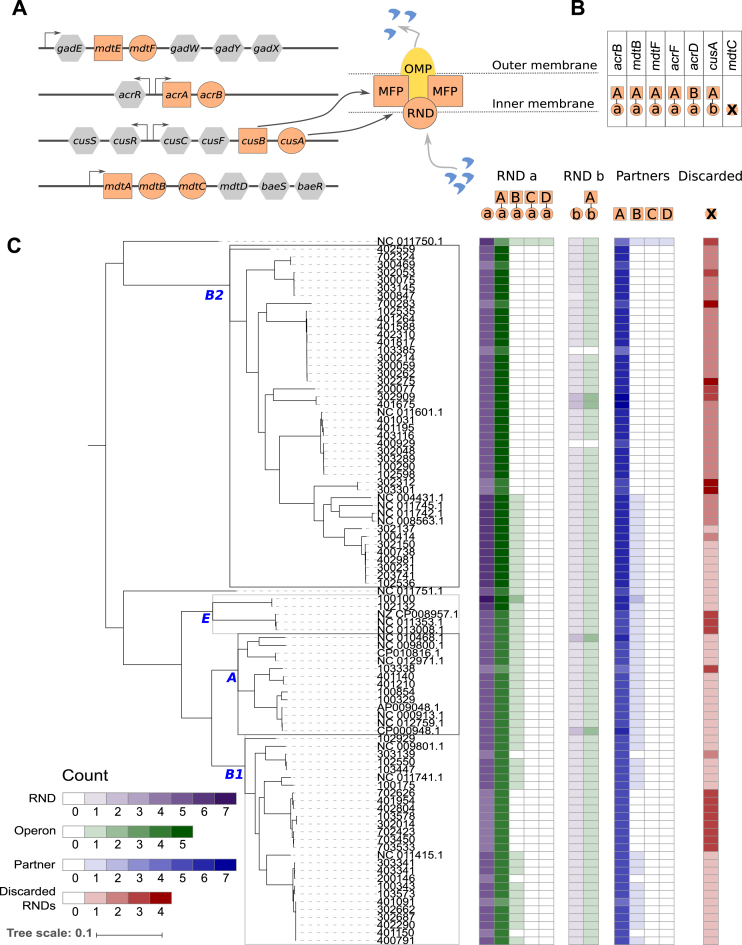
Annotation of RND efflux pumps using SLING. (**A**) Four example operon structures of RND efflux pumps present in *E. coli* K-12 (6). All RND pump proteins HMM share a single conserved profile, represented by a single colour. (**B**) Annotation of RND efflux pumps in *E. coli* K-12 using SLING. (**C**) Description of the diversity of RND efflux pumps in the *E. coli* collection.

Efflux pumps play an important role in multidrug resistance as they confer a mechanism for the efflux of antibiotics (43). One example of this are the resistance-nodulation-division (RND) family of membrane transporters found in Gram-negative bacteria (6,44). RND family pumps consist of three components: an outer membrane protein (OMP), a periplasmic fusion protein (MFP) and an RND pump (Figure 4A). In most cases, the MFP and RND components are found in an operon, whereas the OMP is located in a different location (6). A database of known RND-family Pfam profiles was constructed as above as for TA systems (see Materials and Methods). The RND pump protein is highly conserved and in most instances, the MFP is located upstream of it and transcriptionally coupled to it. However, there are variations to this structure (6). These properties make SLING suitable for the annotation of RND pumps by using the RND pump Pfam profile to identify the conserved gene hit and applying flexible structure requirements on the partner gene.

Seven RND efflux pumps are reported in the literature for *E. coli* K-12 strain W3110 (AP009048.1) (6). Of these, SLING identifies the six RND pumps which fit the structure requirements applied in our analysis: *acrB, cusA, mdtB, acrF, acrD* and *mdtF* (Figure 4B, Supplementary Table S6). Since *mdtC* pump is found downstream to another RND pump, *mdtB*, (Figure 4A) this pump was discarded by SLING as the upstream gene was not in the correct length.

##### *RND efflux pump operons differ in prevalence in* E. coli *collection*

Five unique RND pump operons were identified in this analysis (Figure 4C). These operons consist of two unique RND protein (hit) clusters (a and b) and four partner protein clusters (A-D).

The A partner protein is indeed a MFP and includes all the known MFPs found in *E. coli* K-12 (Figure 4B). It is highly prevalent and found in two different operons, with the two RND pump proteins. The ‘A-a’ operon is ubiquitous, with at least four copies per strain. The ‘A-b’ operon, on the other hand, is found in a single copy in most isolates. The ‘b’ pump corresponds to the *cusA* RND pump in *E. coli* K-12, whereas the ‘a’ pump represents all the other known RND pumps in *E. coli* K-12 (Figure 4B).

The B partner protein is a histidine kinase. This protein maps back to *narQ* gene, found upstream to the *acrD* RND pump in *E. coli* K-12 (6). This operon was lost in specific clades within the B1 and B2 phylogroups. These clades are correlated with the discarded hits, suggesting two events occurred that led to deviation from the expected operon structure in these clades.

Finally, the C and D partner proteins are only observed once and in a single isolate (*E. coli* IAI39, NC_011750.1). Both proteins are short with ‘C’ partner protein 138 aa long and the ‘D’ partner protein 310 aa long. BLAST results of protein ‘C’ against the non-redundant protein sequence database suggest it is a histidine kinase similar to partner protein ‘B’. Protein ‘D’, on the other hand, appears to be a truncated RND pump protein. These appear to be false positives which can be removed by applying more stringent requirements to the permitted length of the partner gene.

## DISCUSSION

**Figure 5.**
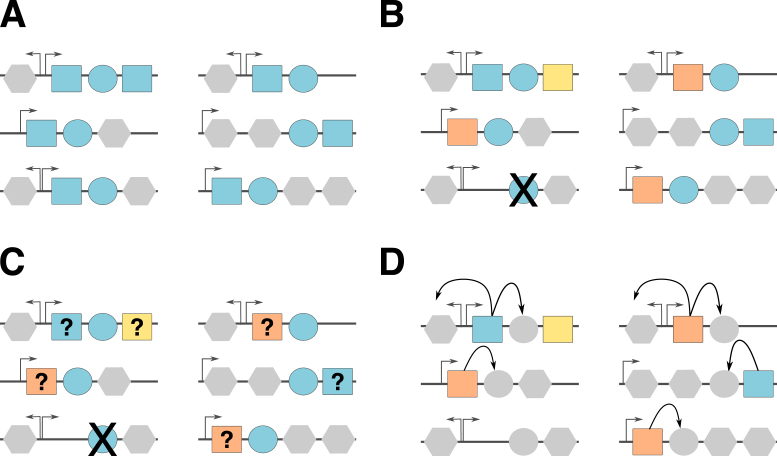
Utility of SLING. (**A**) Search for gene pairs and triplets based on a single conserved gene (circle) and set of rules on the order and orientation of the neighbouring genes (squares). (**B**) Test the defined rules by examining the diversity of the neighbouring genes and identifying gene arrays which deviate from the expected structure. (**C**) Directly identify new genes (squares). ( **D**) Iteratively identify new genes by using the novel neighbour genes (squares) as the input HMM profiles.

We present SLING, an open source tool to identify operons or conserved gene arrays in bacterial datasets by using one of the conserved genes within the array to identify the linked genes which appear in a rule-defined proximity (Figure 5A). SLING enables to test these defined rules by examining the diversity of the neighbouring genes, and by identifying arrays which deviate from the expected structure (Figure 5B). We provide built-in libraries and demonstrate its utility to understand the diversity of a simple two-component operon, TA systems, and a complex operon, the RND efflux pump, in a collection of clinically relevant and reference *E. coli* strains. The utility of SLING is not limited to these operons, and can be easily applied to other important operons or gene pairs such as CRISPR-cas systems, restriction-modification systems, secretion systems, and more. Users may construct HMM libraries and structural requirements in their area of expertise which can be shared with the community by uploading them to the public repository, enabling the extension of the built-in SLING use cases. When searching for an unknown set of linked genes, SLING is a discovery tool which identifies genes linked to the primary gene and the structures in which they are found by applying default flexible structural requirements.

While other tools exist for annotating bacterial genomes for operons, and specifically TA systems, these tools are web-based and do not allow for batch annotations or comparison between different isolates (12,14,38,45–47). Furthermore, they do not allow the addition of custom sequences or domains in the search (3,12,14). This limits the search, and the quality and relevance of the annotation is determined by the quality of the database. Users have to rely on updates to obtain the most up to date results. SLING is flexible and the user can easily append new profiles into its search, enabling easy identification of new and not well studied systems.

We present SLING’s added value in sub-categorising the conserved genes within the gene arrays based on their neighbours. While some genes present high diversity in their possible neighbours, others present low diversity. We would not be able to obtain this understanding by looking at the conserved gene alone. Likewise, by examining the diversity of the neighbouring genes, SLING helps to further sub-categorise the gene combinations according to varying indications of these arrays being lost or gained.

Finally, as SLING relies on a single gene for its search, it can be used to search for novel genes either directly, by looking at the partner genes identified (Figure 5C), or indirectly, but constructing HMM profiles of the newly identified partner genes and iteratively using these as the conserved gene (Figure 5D).

## DATA AVAILABILITY

SLING is available to download from the GitHub repository https://github.com/ghoresh11/sling. The alignment and tree files are available under https://doi.org/10.6084/ m9.figshare.5977783.v1. The accession numbers of all strains used in this paper are available in the Supplementary Tables S2 and S3.

## Supplementary Material

Supplementary Data
