# Supplementary material for: SLING: a tool to search for linked genes in bacterial datasets
Source: Nucleic Acids Res. 2018 Aug 16;46(21):e128. doi: 10.1093/nar/gky738 (PMC6265476; doi:10.1093/nar/gky738)
Supplement: Supplementary Data [file gky738_supplemental_files.zip › Horesh_sup_figures.pdf]

A

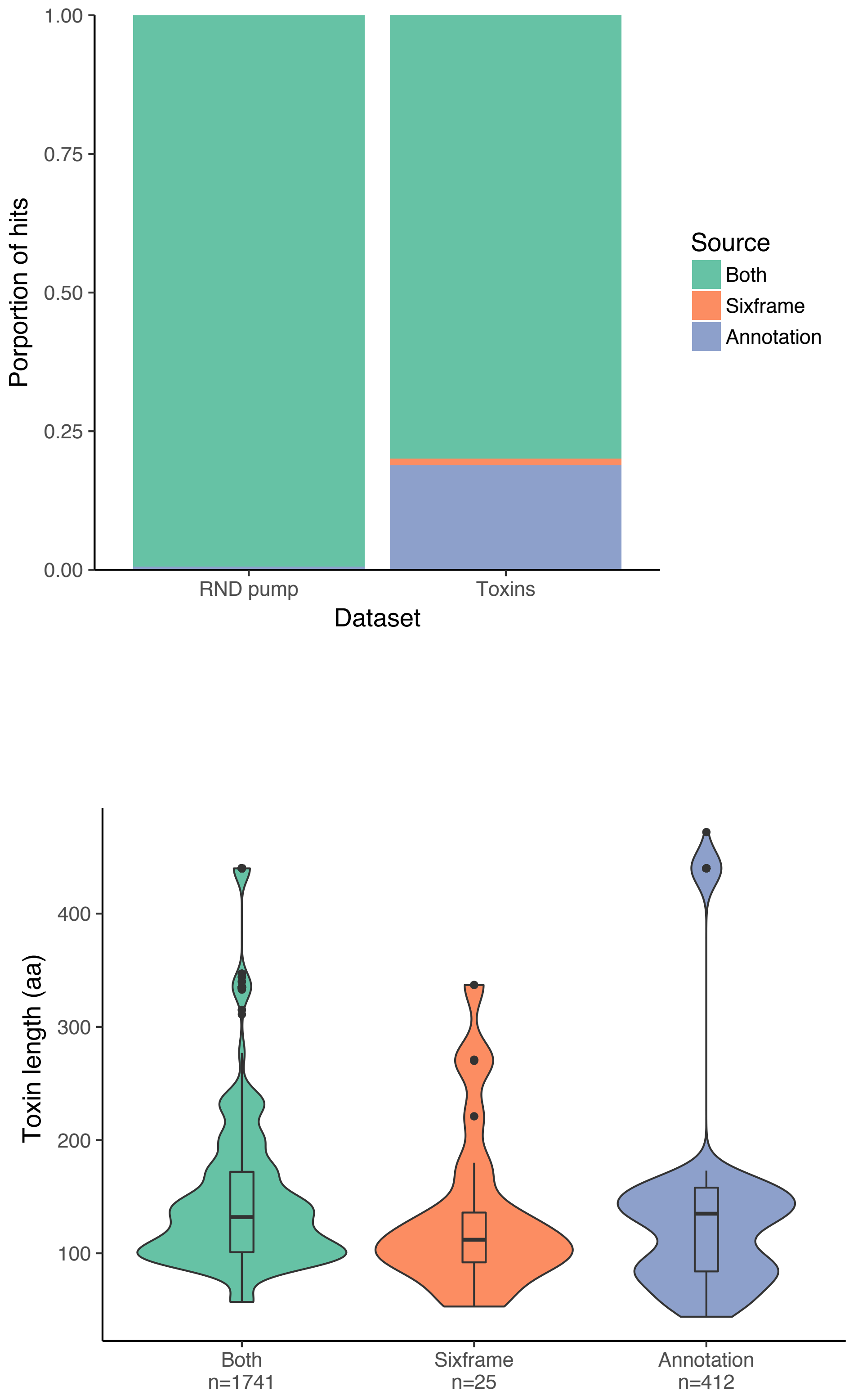

B

Figure S1: Hits identified by SLING using six-frame translated CDSs and Prokka annotation CDSs. **A** Porpotion of hits identified only in six-frame CDSs (orange), annotation CDSs (blue) or in both (green). **B** Length of toxin hits identified only in six-frame CDSs, annotation CDSs and in both.

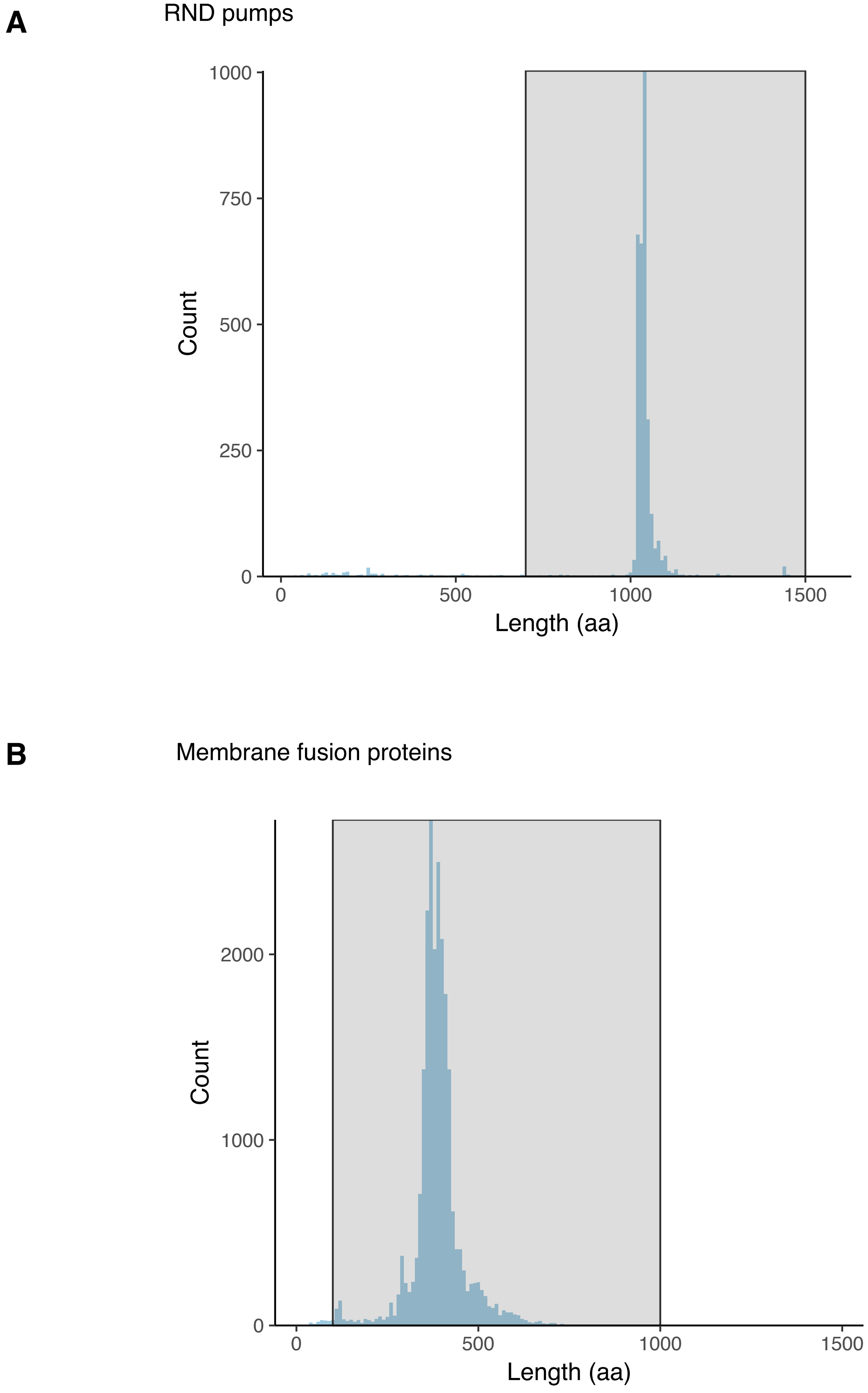

Figure S2: Length distribution of all RND pump protein (**A**) and membrane fusion protein (**B**) sequences downloaded from Uniprot. The gray box is drawn around the minimum and maximum length requirements applied in this paper.

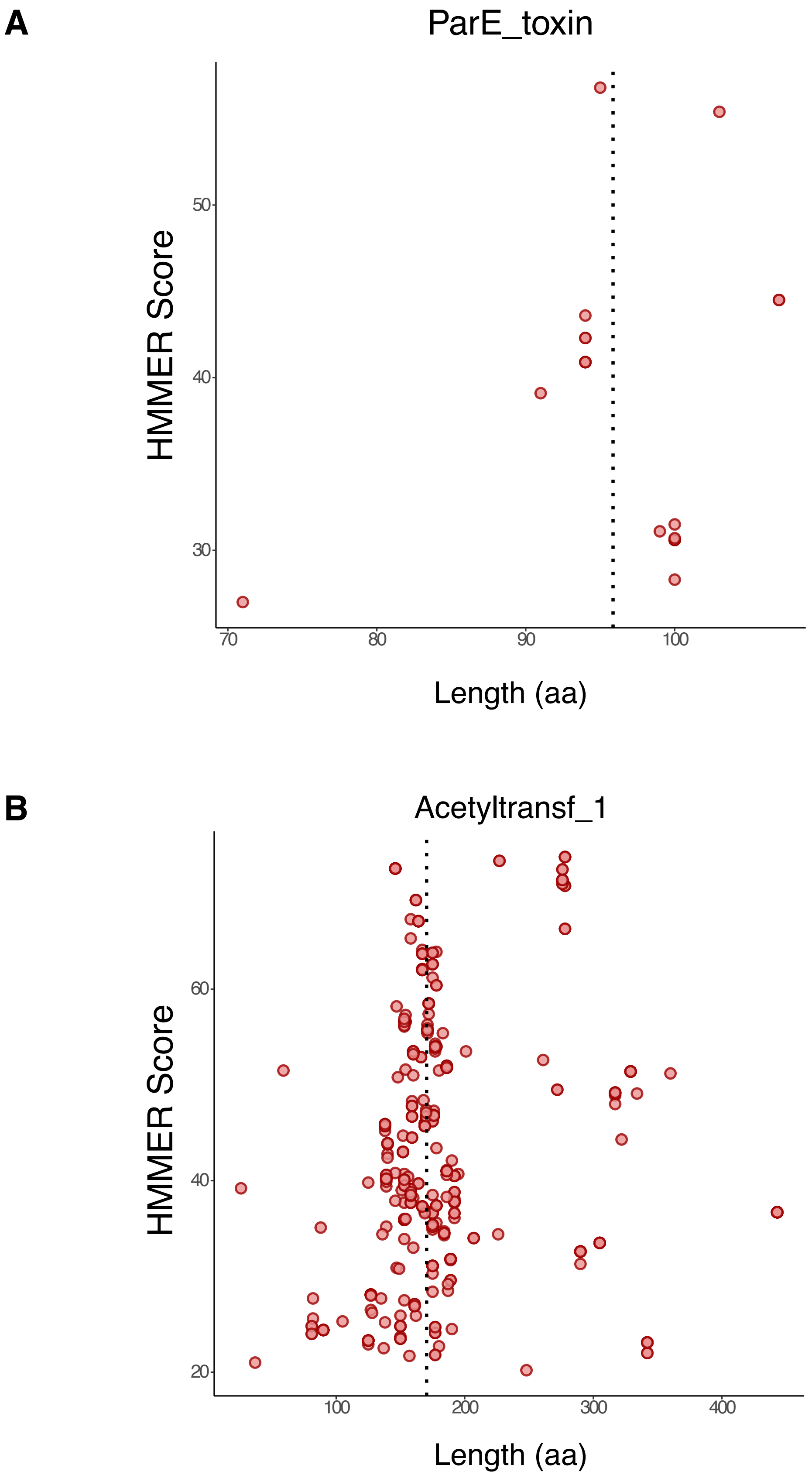

Figure S3: Example of specific (**A**) versus non-specific (**B**) toxin profile. Red dots are a single hit of an CDS containing the profile in an example dataset of 33 *Klebsiella* spp. genomes and plasmids. Dotted line is the average length of ORFs containing the profile in TADB. Specific toxin profile hits tend to fall around the average TADB length. Non-specific profiles hits are very abundant and variable in length.

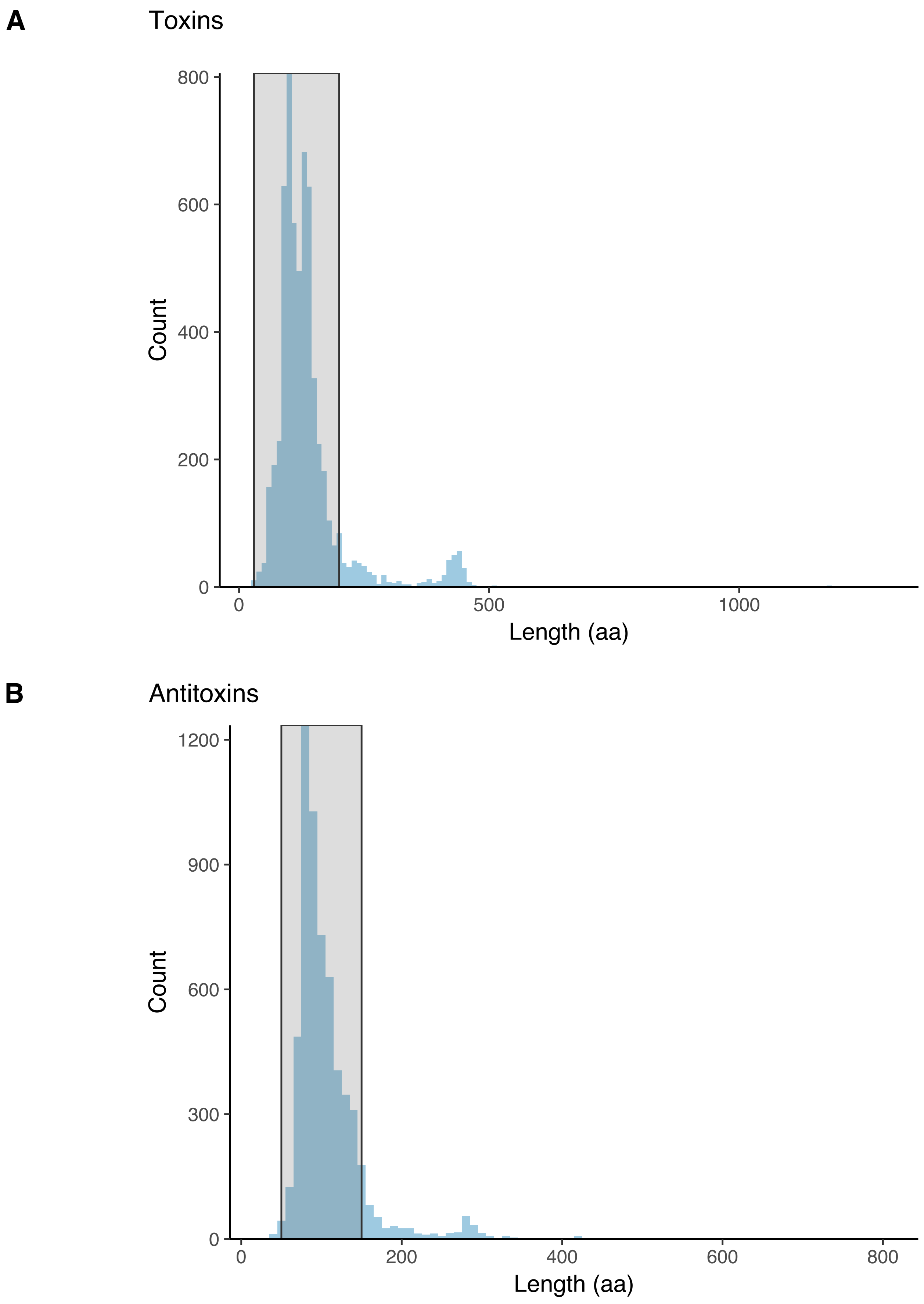

Figure S4: Length distribution of all toxin (**A**) and antitoxin (**B**) sequences downloaded from TADB. The gray box is drawn around the minimum and maximum length requirements applied in this paper.

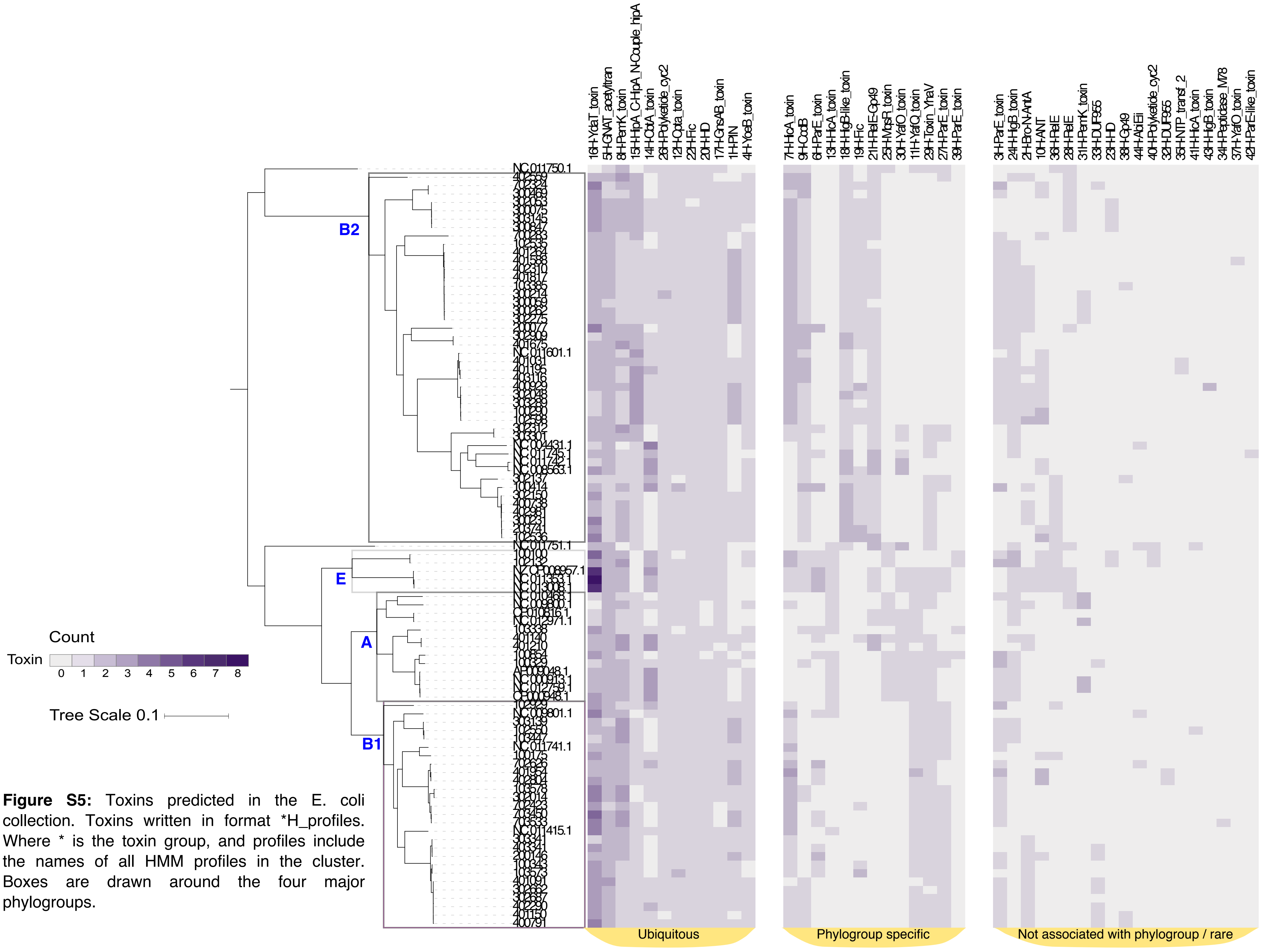

Figure S5: Toxins predicted in the E. coli collection. Toxins written in format *H_profiles. Where * is the toxin group, and profiles include the names of all HMM profiles in the cluster. Boxes are drawn around the four major phylogroups.

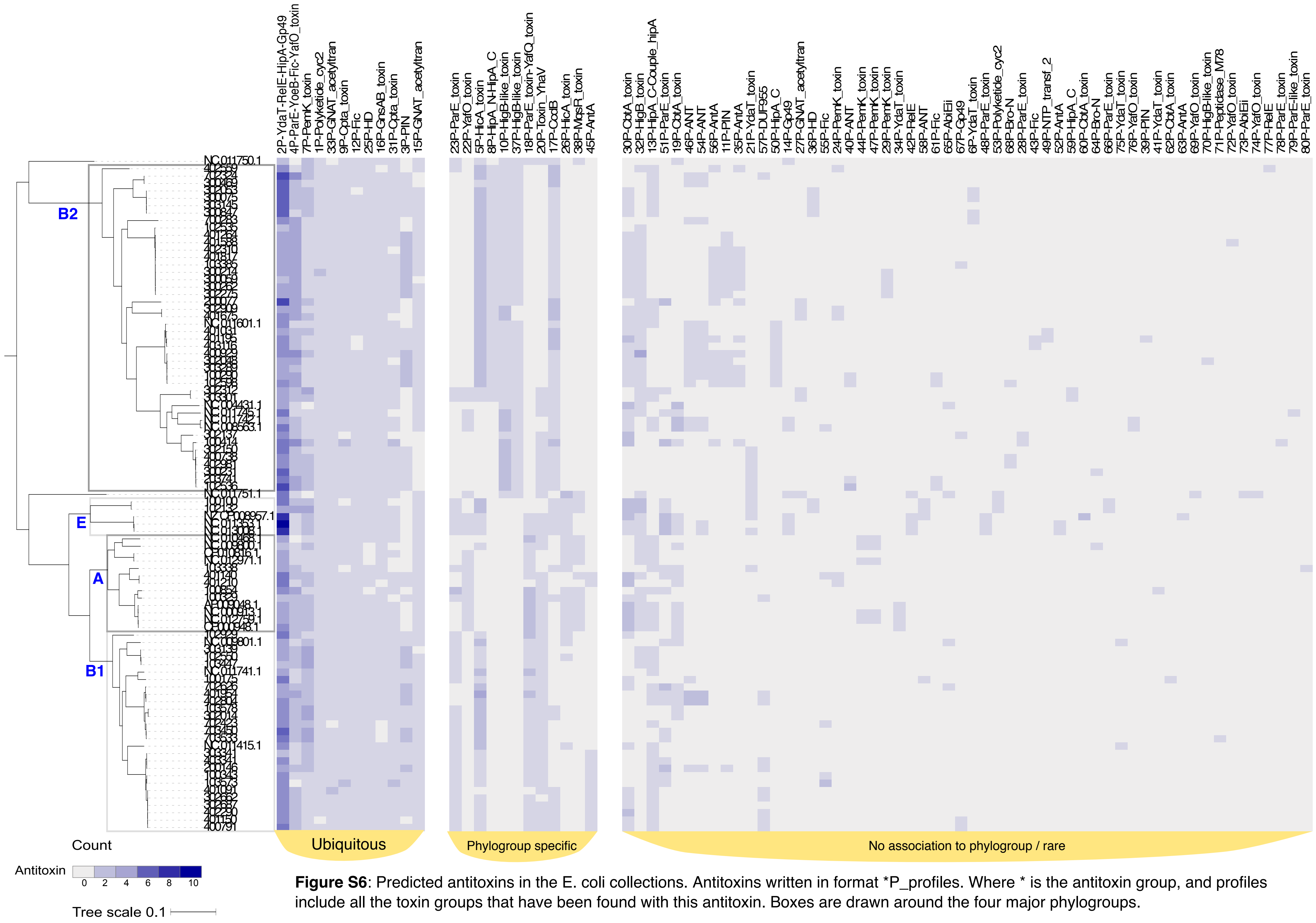

Figure S6: Predicted antitoxins in the E. coli collections. Antitoxins written in format *P_profiles. Where * is the antitoxin group, and profiles include all the toxin groups that have been found with this antitoxin. Boxes are drawn around the four major phylogroups.

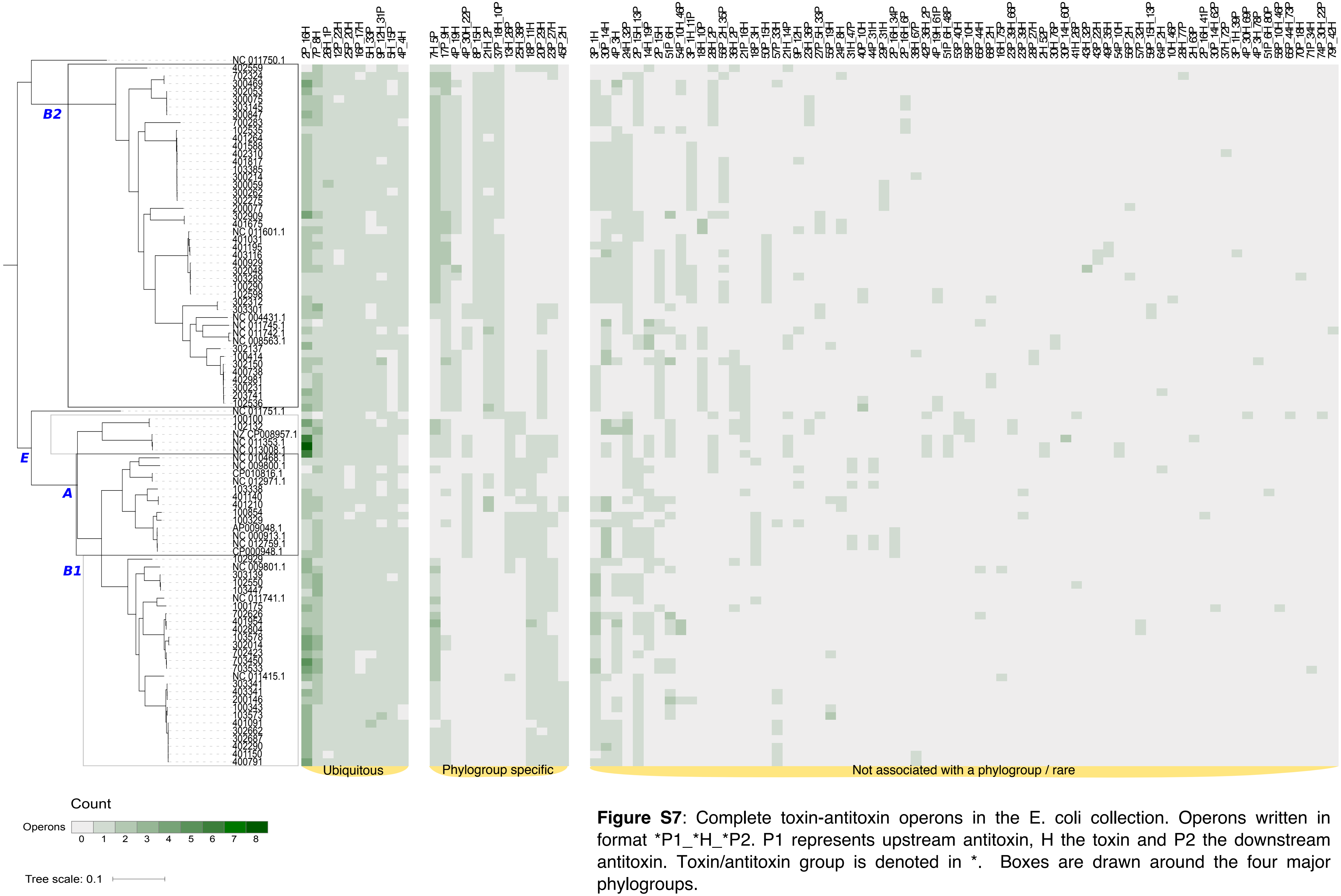

Figure S7: Complete toxin-antitoxin operons in the E. coli collection. Operons written in format *P1_*H_*P2. P1 represents upstream antitoxin, H the toxin and P2 the downstream antitoxin. Toxin/antitoxin group is denoted in *. Boxes are drawn around the four major phylogroups.
